# Anti-interleukin-6 receptor antibody treatment ameliorates postoperative adhesion formation

**DOI:** 10.1038/s41598-019-54175-1

**Published:** 2019-11-26

**Authors:** Naoki Uyama, Hiroko Tsutsui, Songtao Wu, Koubun Yasuda, Etsuro Hatano, Xian-Yang Qin, Soichi Kojima, Jiro Fujimoto

**Affiliations:** 1 Department of Surgery, Hyogo College of Medicine, Nishinomiya, Hyogo, Japan; 2 Department of Immunology, Hyogo College of Medicine, Nishinomiya, Hyogo, Japan; 3 Liver Cancer Prevention Research Unit, RIKEN Center for Integrative Medical Sciences, Wako, Saitama, Japan

**Keywords:** Experimental models of disease, Molecular medicine

## Abstract

Postoperative adhesion formation often ruins the quality of life or is an obstacle to illnesses with curative operation such as cancer. Previously we demonstrated that interferon-γ-promoted fibrin deposition drove postoperative adhesion formation. However, its underlying cellular and molecular mechanisms remain poorly understood. We found that myofibroblasts of the adhesion predominantly expressed signature molecules of mesothelial cells that line the serosa. Microarray analysis revealed IL-6 as a key underlying player, supported by elevated IL-6 levels in the peritoneal fluid of post-laparotomy human subjects. Injured serosa of cecum-cauterized mice also exhibited induction of *Il6*, which was followed by *Tnf*, concomitant with rapid accumulation of neutrophils, substantial population of which expressed TGF-β1, a master regulator of fibrosis. Besides, neutrophil-ablated mice showed reduction in induction of the adhesion, suggesting that TGF-β1^+^neutrophils triggered the adhesion. Human neutrophils expressed *TGFB*1 in response to TNF-*α* and *TNF* in response to IL-6. Moreover, anti-IL-6 receptor monoclonal antibody abrogated neutrophil recruitment and adhesion formation. Thus, IL-6 signaling represents a potential target for the prevention of postoperative adhesions.

## Introduction

Abdominal adhesions form in response to peritoneal trauma that can occur during surgery^1,2^. Postoperative adhesions, which develop in approximately 50–85% of patients who have undergone abdominal surgery, often result in severe complications, including intestinal obstruction, female infertility, chronic pain, and contraindication of surgery for future abdominal illnesses such as cancer^3,4^. Treatment of associated morbidities costs up to $1.3 billion annually in the United States alone^5^.

We previously reported that levels of the plasminogen activator inhibitor type 1 (PAI-1), a potent inhibitor of fibrinolysis^6^, were increased in injured ceca and remnant liver in a manner dependent on interferon gamma (IFN-γ) following cecal cauterization and partial hepatectomy, respectively. Furthermore, this increase was required for the development of postsurgical adhesions^7,8^. However, the cellular and molecular mechanisms underlying the inflammatory and pro-fibrous responses have remained poorly understood.

The surface of every peritoneal organ, such as the intestine and the liver, is lined with a single layer of mesothelial cells^9^. Here, we investigated mesothelial cells as a source of collagen production in postoperative adhesions, explored molecular mechanisms of adhesion using microarray analysis, and identified interleukin 6 (IL-6) signaling as a novel therapeutic target for preventing postoperative adhesion formation.

## Results

### Activation of the procoagulant response upon cecal cauterization

We utilized immunohistological staining to investigate fibrin and collagen deposition following cecal cauterization in mice. Dense fibrin deposition and poor collagen production were observed 12 h following the procedure. However, the injured serosa showed signs of robust collagen deposition and weaker fibrin deposits on day 7 (Supplementary Fig. 1a). Similarly, intraperitoneal exudates obtained from first-time laparotomy patients during the operation exhibited dense fibrin deposition, but poor collagen formation (Supplementary Fig. 1b). In contrast, specimens resected from adhesion tissue of previous laparotomy patients exhibited dense collagen, but poor fibrin, deposition (Supplementary Fig. 1b). Thus, fibrotic changes likely develop subsequently to fibrin deposition in the injured serosa.

### Signatures of mesothelial cells in myofibroblasts in the fibrous adhesion band

**Figure 1:**
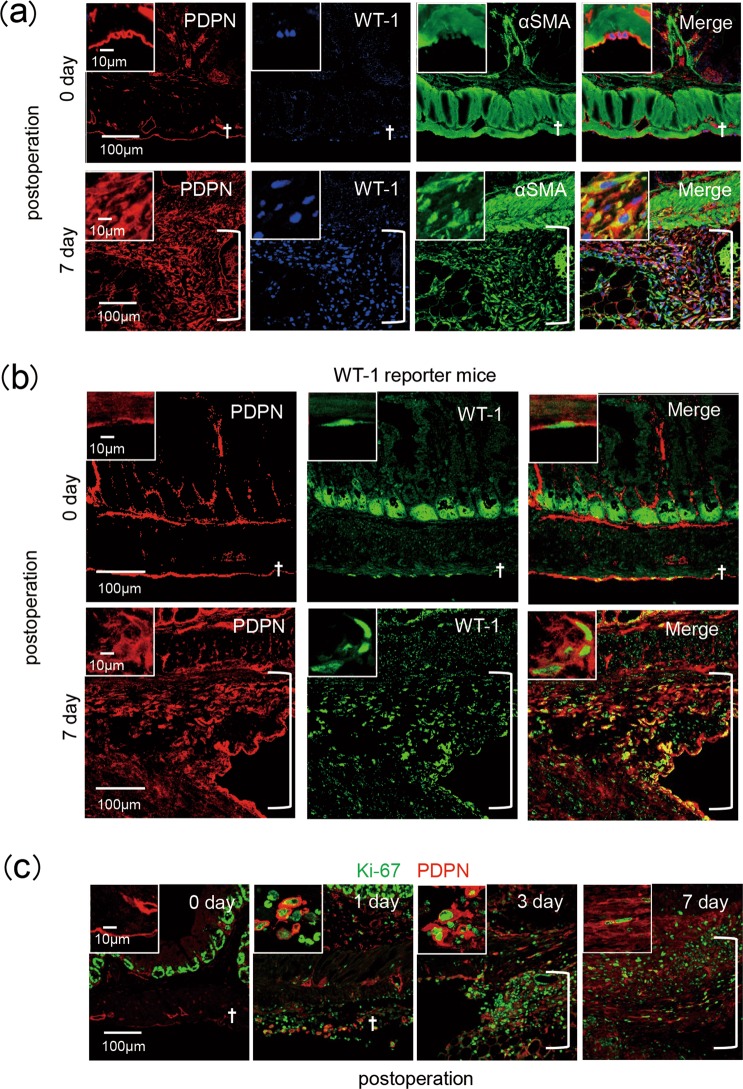
Mesothelial cell transdifferentiation into myofibroblasts in the adhesion band. (**a,c**) Cecum lesions were sampled at the indicated time points following cecum cauterization in wild-type mice. (**b**) Immunostaining of the adhesion lesion after cecum cauterization of Wilms tumor protein (WT-1) reporter mice. Each experimental group contained 3–5 mice, with two independent experiments performed. Representative photos are shown. Data at 0 day postoperation indicated those in untreated control mice. Dagger symbols indicate serosa; open brackets indicate adhesion lesions. PDPN, podoplanin; αSMA, α smooth muscle actin.

Myofibroblasts originate from multiple cellular sources, including fibroblasts, vascular pericytes, epithelial cells, and mesothelial cells^10,11^. We investigated whether adhesion-associated myofibroblasts originate from mesothelial cells lining the peritoneum. Major alpha smooth muscle actin (αSMA)-positive myofibroblasts in the adhesion band expressed podoplanin as well as WT-1 (Fig. 1a), suggesting transdifferentiation of mesothelial cells into myofibroblasts during adhesion formation. This possibility was underscored by analysis of WT-1 reporter mice: a single-cell layer of WT-1^+^ mesothelial cells was present on the cecal serosa under normal conditions, whereas an accumulation of cells co-expressing WT-1 and podoplanin was observed in the adhesion band (Fig. 1b). Furthermore, the podoplanin-expressing cells were positive for the Ki-67 protein (Fig. 1c), indicating proliferation of mesothelial cell-derived myofibroblasts in the adhesion. Consistent with this, Tsai *et al*. recently demonstrated the mesothelial cells as a major cell type of adhesion-associated myofibroblasts by clonal analysis and lineage tracing^12^. These results strongly suggested that surgical injury can convert cecal mesothelial cells into myofibroblasts to generate the adhesion band.

### Sequential induction of inflammatory and pro-fibrous responses

We performed microarray analysis to identify key molecules and signaling pathways associated with adhesion. Transcriptional profiling was performed using biological triplicates of control cecum tissues and those isolated at 3 different time points during adhesion formation (3, 12, and 72 h after cecal cauterization). Unsupervised principal component analysis (PCA) was first applied to the transcriptomic data comprising 56,743 detected probes to obtain a global view of the dynamic expression changes during cecum adhesion formation (Supplementary Fig. 2a). The PCA score plot (PC 1 vs. 2) showed strong and reproducible time-dependent changes in gene expression patterns of cecum tissues during adhesion formation. According to the first component (PC1), representing 28.6% of the total variance, significant changes in gene expression during cecum adhesion formation were observed as early as 3 h following cauterization, returning to baseline levels following 72 h.

To gain greater insights into the biological processes underlying cecum adhesion formation, we analyzed the differentially expressed genes at each time point relative to the sham controls (3,957, 4,962, and 4,370 genes at 3, 12, and 72 h, respectively) using the Ingenuity® Pathway Analysis (IPA) program. The biological function analysis revealed activation of immune cell chemotaxis, as well as of cellular migration and movement, in the cecum tissues as early as 3 h after cauterization. In addition, canonical pathway analysis uncovered the enrichment of genes involved in the IL-6 signaling pathway in post-cauterization cecum tissues isolated at this time point (Supplementary Fig. 2b). Upstream regulator analysis identified IL-6 as a master regulator of cecum adhesion formation (Supplementary Fig. 2c). Furthermore, an IL-6-dependent regulatory network generated by knowledge-based pathway analysis in the IPA platform highlighted the importance of IL-6 in governing cell migration (Supplementary Fig. 2d).

**Figure 2:**
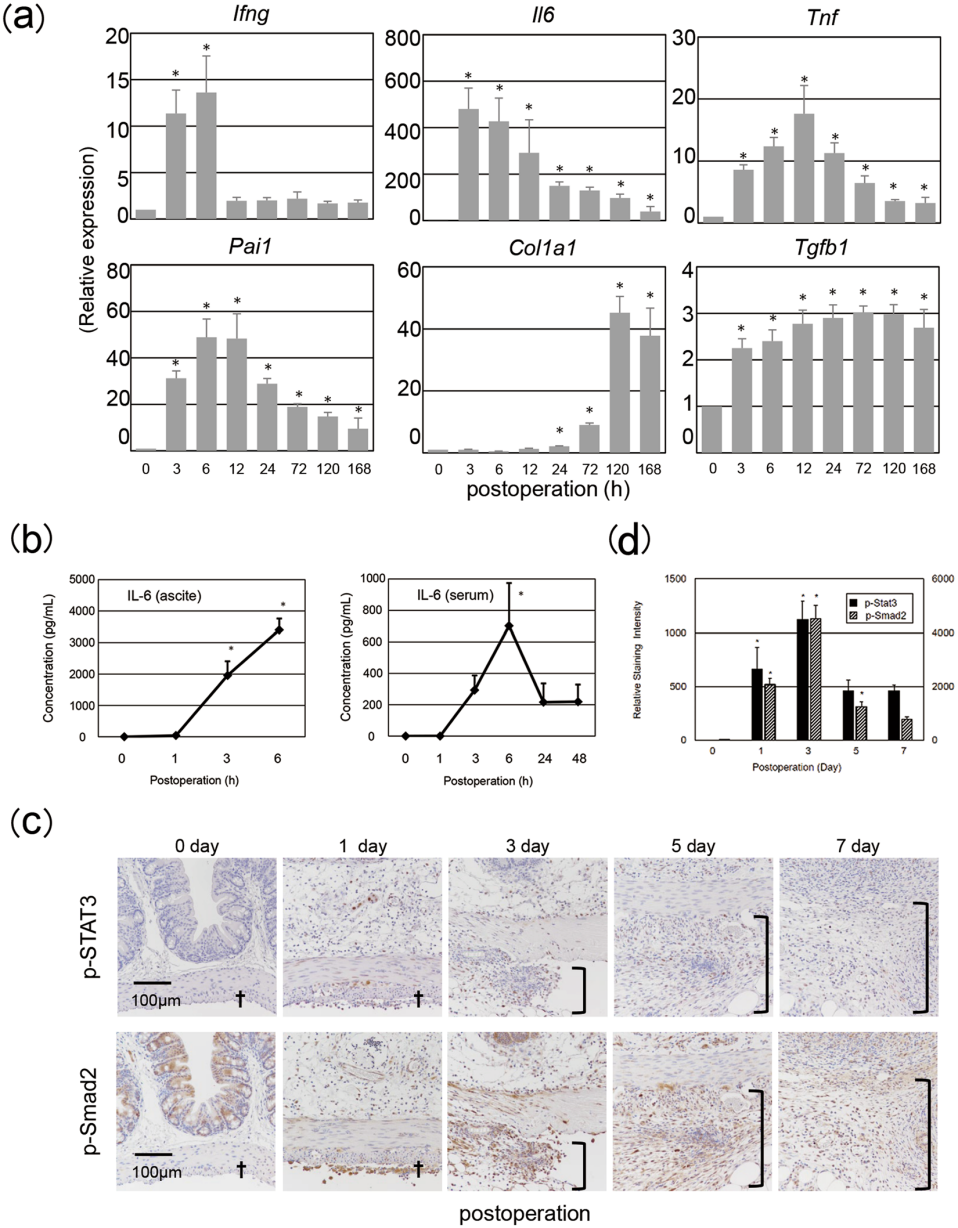
Proinflammatory responses prior to pro-fibrotic alterations. (**a,c**) Cecum lesions were sampled from each experimental group (3–5 mice/group) at the indicated time points post operation, followed by qRT-PCR assessment of expression of proinflammatory cytokine and pro-fibrotic molecule genes (**a**) and by immunostaining of phosphorylated transcription factors with ImageJ analysis data (**c,d**) *Ifng*, interferon γ; *Il6*, interleukin-6; *Tnf*, tumor necrosis factor-α; *Pai1*, plasminogen activator inhibitor type 1; *Col1a1*, collagen 1α1; *Tgfb1*, tumor growth factor- β1. (**b**) Peritoneal fluid and serum were sampled from human patients at the indicated time points following the beginning of laparotomy, and IL-6 levels were measured using ELISA. Representative photos are shown. Data at 0 day or hour postoperation indicated those in untreated control mice. Data are shown as mean ± SD. *p < 0.05 (Dunnett’s test). Daggers indicate serosa; open brackets indicate adhesion lesions. All experiments were independently repeated twice.

Based on these results, we hypothesized that damage-associated molecular patterns (DAMPs) in cauterized ceca activate pattern recognition receptors to stimulate production of proinflammatory cytokines such as IL-6 and tumor necrosis factor alpha (TNF-α), which in turn activate transforming growth factor beta 1 (TGF-β1) production, leading to fibrous adhesion formation. To test this hypothesis, we measured levels of mRNA encoding proinflammatory cytokines, pro-fibrotic factors, and fibrinolysis-associated proteins in the injured ceca sampled at the indicated time points. Consistent with the microarray data (Supplementary Fig. 2), levels of *Ifng* and *Il6* transcripts were immediately elevated, followed by increases in abundance of mRNA encoding TNF-α and PAI-1. Up-regulation of *Tgfb*1 and *Col*1*α1* followed the elevated production of these proinflammatory and anti-fibrinolytic transcripts (Fig. 2a). Consistent with this, levels of the respective proteins were also elevated in the peritoneal fluid (ascites) (Supplementary Fig. 3). Similarly, human peritoneal fluid and serum sampled starting at 3 h post laparotomy contained elevated concentrations of IL-6 (Fig. 2b).

To investigate whether IL-6 exerts its biological action in the injured cecum, we examined activation of its transcription factor, signal transducer and activator of transcription 3 (STAT3). STAT3 was substantially phosphorylated in the injured ceca following surgery (Fig. 2c), suggesting that IL-6 and other STAT3-activating cytokines generated at the injured sites potentially activate the IL-6 receptor (IL-6R)/glycoprotein 130 (gp130)-mediated signaling pathway. In addition, phosphorylation of Smad2, an event downstream of TGF-β1 signaling, was observed in the injured cecal serosa on day 1 and in the adhesion starting on day 3 (Fig. 2c), suggesting the involvement of active TGF-β1 in fibrous adhesion formation, as well as in the serosa of the injured ceca. Overall, these results highlighted the induction and signaling of proinflammatory cytokines and pro-fibrotic molecules during the adhesion formation process.

### Accumulation of TGF-β1-producing neutrophils in the injured serosa

**Figure 3:**
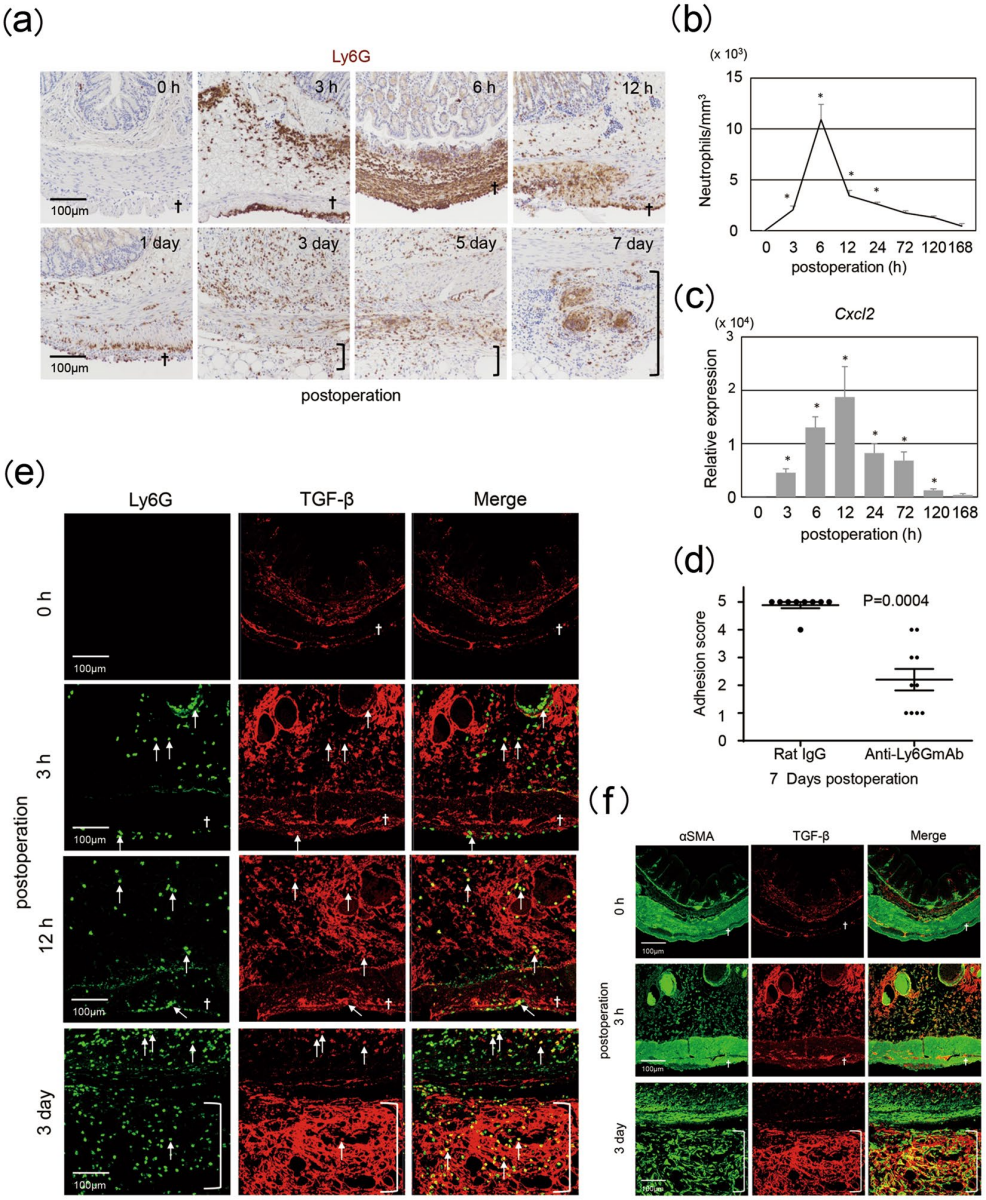
Importance of neutrophils in adhesion formation. Cecum lesions were sampled from wild-type mice at the indicated time points following operation for the analysis of neutrophil accumulation by staining for Ly6G (**a**), Ly6G and TGF-β1 (**e**), or αSMA and TGF-β1 (**f**), for counting Ly6G^+^ cells (**b**), and for quantitation of *Cxcl2* expression (**c**). Neutrophils were depleted in wild-type mice using treatment with anti-Ly6G antibodies. Adhesion scores were evaluated 7 days following cecum cauterization in neutrophil-ablated mice (**d**). Each experimental group contained 3–5 mice, with two independent experiments performed. Data at 0 hour postoperation indicated those in untreated control mice. Data are shown as mean ± SD. *p < 0.05 (Dunnett’s test for Fig. 2b,c, Student’s paired t-test for Fig. 2d). Representative photos are shown. Daggers indicate serosa; open brackets indicate adhesion lesions.

We next assessed the types of cells that migrated into the injured site. Lymphocyte antigen 6 complex, locus G-positive (Ly6G^+^) neutrophils accumulated at the site, peaking in numbers around 6 h post operation (Fig. 3a,b). In contrast, obvious migration of T cells, B cells, or macrophages was not observed (Supplementary Fig. 4). Neutrophil accumulation began to gradually diminish at 12 h following the procedure (Fig. 3a,b). Next, we investigated how neutrophils were accumulated in the injured site. It is well documented that neutrophils express chemokine receptor CXCR2 and can migrate into the target sites in a manner dependent on the density gradient of CXCR2 ligands, such as CXCL1, CXCL2, and CXCL5^13^. Therefore, we investigated the kinetics of these CXCR2 ligands and found that *Cxcl2*, but not *Cxcl1* or *Cxcl5*, was significantly induced postoperation. Expression levels of *Cxcl2* increased at the injury site, peaking at 12 h (Fig. 3c). To evaluate the contribution of neutrophils to adhesion formation, we depleted these cells by administration of an anti-Ly6G monoclonal antibody 1 day prior to surgery^14^. Neutrophil-ablated mice showed reduced adhesion formation upon cecal cauterization (Fig. 3d).

We next sought to examine the role of neutrophils in adhesion formation. TGF-β1 is a well-established master inducer of fibrosis^15–18^. It was reported that both active and total TGF-β1 concentrations were more than twice as high as unaffected peritoneoum and that blockade of both TGF-β1 and TGF-β2 using neutralizing antibodies prevented abdominal adhesion formation^19,20^. These reports strongly suggested the importance of TGF-β1 for the adhesion formation. We accordingly observed phosphorylated Smad2 in the injured serosa following adhesion formation (Fig. 2c). This observation allowed us to investigate whether neutrophils recruited to the injured site produce TGF-β1. Immunostaining revealed the presence of TGF-β1^+^Ly6G^+^ neutrophils in the injured serosa and adjacent submucosa at 3 and 12 h post procedure (Fig. 3e). TGF-β1^+^ neutrophils accumulated at the adhesion site by day 3 (Fig. 3e). On day 3, αSMA^+^ myofibroblasts, likely of mesothelial cell origin (Fig. 1), produced similar levels of TGF-β1 (Fig. 3f). Taken together, these results suggested that neutrophils migrating to the injured serosa likely produce TGF-β1, thereby inducing collagen production by mesothelial cell-derived myofibroblasts and promoting adhesion formation.

### Crosstalk between neutrophils and mesothelial cells during adhesion formation

**Figure 4:**
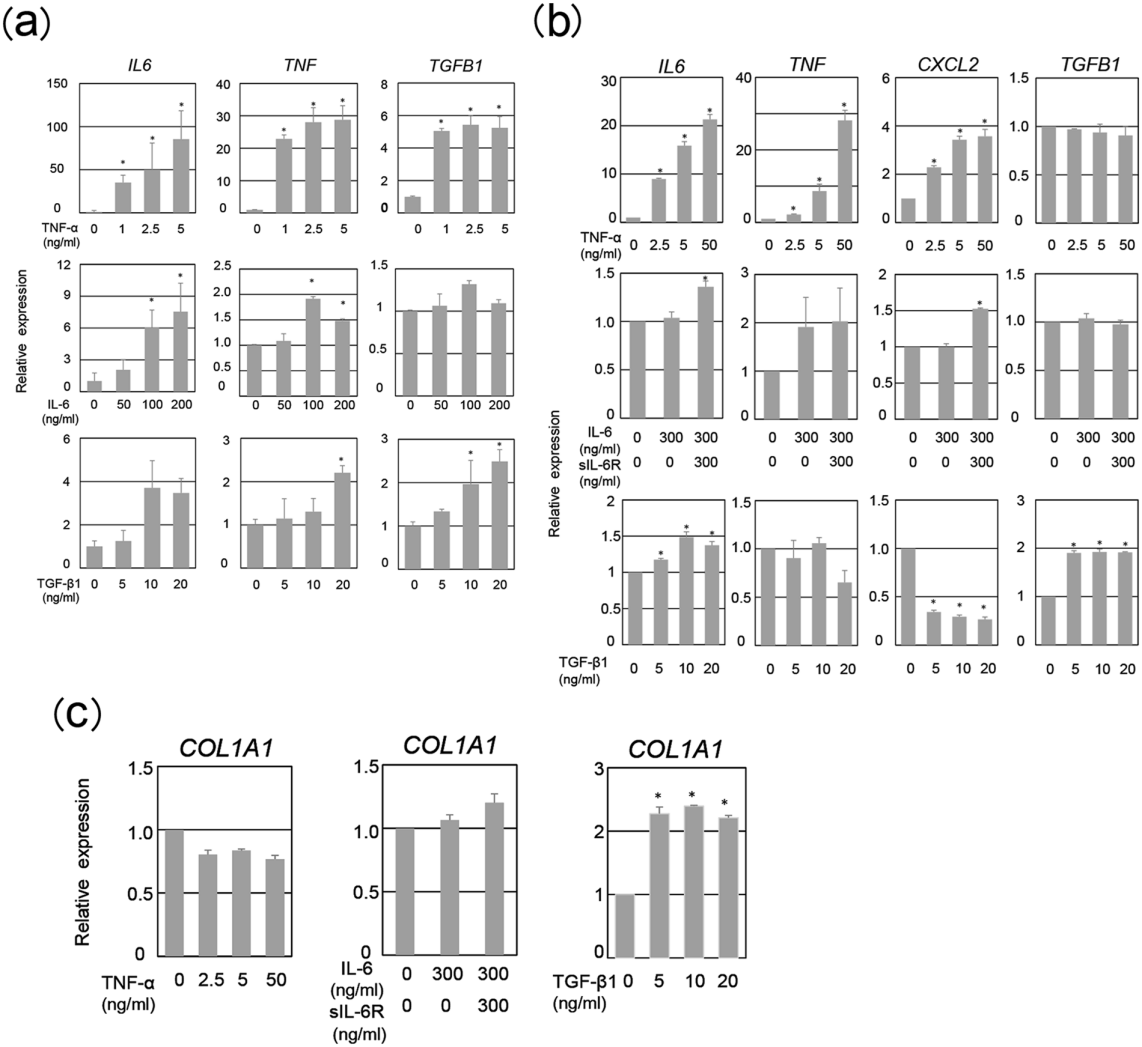
Production of pro-fibrotic molecules by human neutrophils in response to proinflammatory cytokines. Expression of *IL6*, *TNF*, and *TGFB1* was determined in human neutrophils stimulated with TNF-α, IL-6, or TGF-β1 using qRT-PCR (**a**). Human mesothelial cells (MeT5A cells) were incubated with TNF-α, IL-6 plus soluble IL-6Rα (sIL-6Rα), TGF-β1, or IFN-γ, followed by measurement of *IL6* (**b**), *TNF* (**b**), *CXCL2* (**b**), *TGFB1* (**b**), or *COL1A1* expression by qRT-PCR (**c**). Three independent experiments were performed. Data are shown as mean ± SEM. *p < 0.05 (Dunnett’s test).

We sought to determine the molecular mechanism driving TGF-β1 production by neutrophils. Neutrophils in the injured serosa might be affected by the cytokine milieu of the sites. *Il6* and *Tnfα* induction started at 3 h at latest postoperation (Fig. 2a), at which time point and later neutrophils migrated (Fig. 3a,b). This let us to hypothesize that these proinflammatory cytokines trigger TGF-β1 production in neutrophils. To test this, we stimulated human neutrophils with IL-6 and TNF-α *in vitro* and measured *TGFB1* transcript levels. Neutrophils expressed receptors for IL-6, both IL-6-binding IL-6R and IL-6-signaling gp130 and for TNF-α (data not shown)⊡ TNF-α, but not IL-6, induced *TGFB1* in neutrophils (Fig. 4a).

We examined actions of these proinflammatory cytokines on human mesothelial cell line MeT5A cells. In contrast to neutrophils, MeT5A cells lacked the IL-6-binding receptor IL-6Rα but expressed gp130 (Supplementary Fig. 5 and Supplementary Fig. 10). IL-6 can either bind to the membrane-bound IL-6Rα (classical signaling) and to soluble forms of the IL-6Rα (sIL-6Rα) (trans-signaling)^21,22^. Thus, we stimulated MeT5A cells with IL-6 in the presence of sIL-6Rα, due to the stable levels of sIL-6Rα detected in the peritoneal fluid post operation (Supplementary Fig. 3). IL-6/sIL-6Rα did not induce *TGFB1* in Met5A cells. TNF could not induce *TGFB1* in MeT-5A cells (Fig. 4b). Thus, TNF-α could activate *TGFB1* expression only in neutrophils but not mesothelial cells, and IL-6 signaling failed to induce *TGFB1* in either cell types.

We wanted to know whether levels of TNF, a key TGF-β1-inducer (Fig. 4a), were up-regulated by the proinflammatory cytokines. Both neutrophils and MeT5A cells increased *TNF* expression in response to TNF-α (Fig. 4a,b). IL-6 signaling induced *TNF* in neutrophils but not MeT5A cells (Fig. 4a,b). Accordingly, although IL-6 signaling could not directly induce *TGFB1*, IL-6 signaling might contribute to the neutrophilic *TGFB1* induction via TNF-α induction. Immunofluorescence study revealed TNF-α production in both cell types *in vivo* (Supplementary Fig. 6).

Consistent with the previous reports^23,24^, TGF-β1 induced *TGFB1* and *COL1A1* expression in MeT5A cells (Fig. 4b,c). TGF-β1 also induced *TGFB1* in neutrophils. This might implicate the presence of a positive circuit for pro-fibrotic cues in mesothelial cells and possibly in neutrophils as well. These data might suggested that TGF-β1 produced by neutrophils might stimulated mesothelial cells to transdifferentiated into myfobroblasts and to produce robust TGF-β1.

Both IL-6 signaling and TNF-α could activate *CXCL2* in MeT5A cells. In contrast, TGF-β1 strongly dampened *CXCL2* expression in MeT5A cells (Fig. 4b), suggesting that TGF-β1 that produced at day 1 and later (Fig. 2a) potentially prevented neutrophil accumulation via dampening *Cxcl2* (Fig. 3a–c).

*IL-6* was up-regulated by IL-6 signaling and TNF, and even TGF-β1 in neutrophils and MeT5A cells (Fig. 4a,b). Immunofluorescence study revealed IL-6 production in both cell types *in vivo* (Supplementary Fig. 7).

Although IL-6 by itself could not induce *TGFB1* in either neutrophils or mesothelial cells (Fig. 4), IL-6 activated the expression of *CXCL2* and *TNF* in mesothelial cells and neutrophil, respectively. The former IL-6-induced CXCL2 might be involved in the accumulation of neutrophils, and the latter IL-6-induced TNF might contribute to TGF-β1 production in neutrophils. Taken together, these results suggest that the crosstalk between mesothelial cells and neutrophils might induce early TGF-β1 in neutrohils, which initiates the differentiation of mesothelial cells into myofibroblasts, an essential step for adhesion formation.

### Anti-IL-6Rα monoclonal antibody (mAb) treatment protects against postsurgical adhesion formation

**Figure 5:**
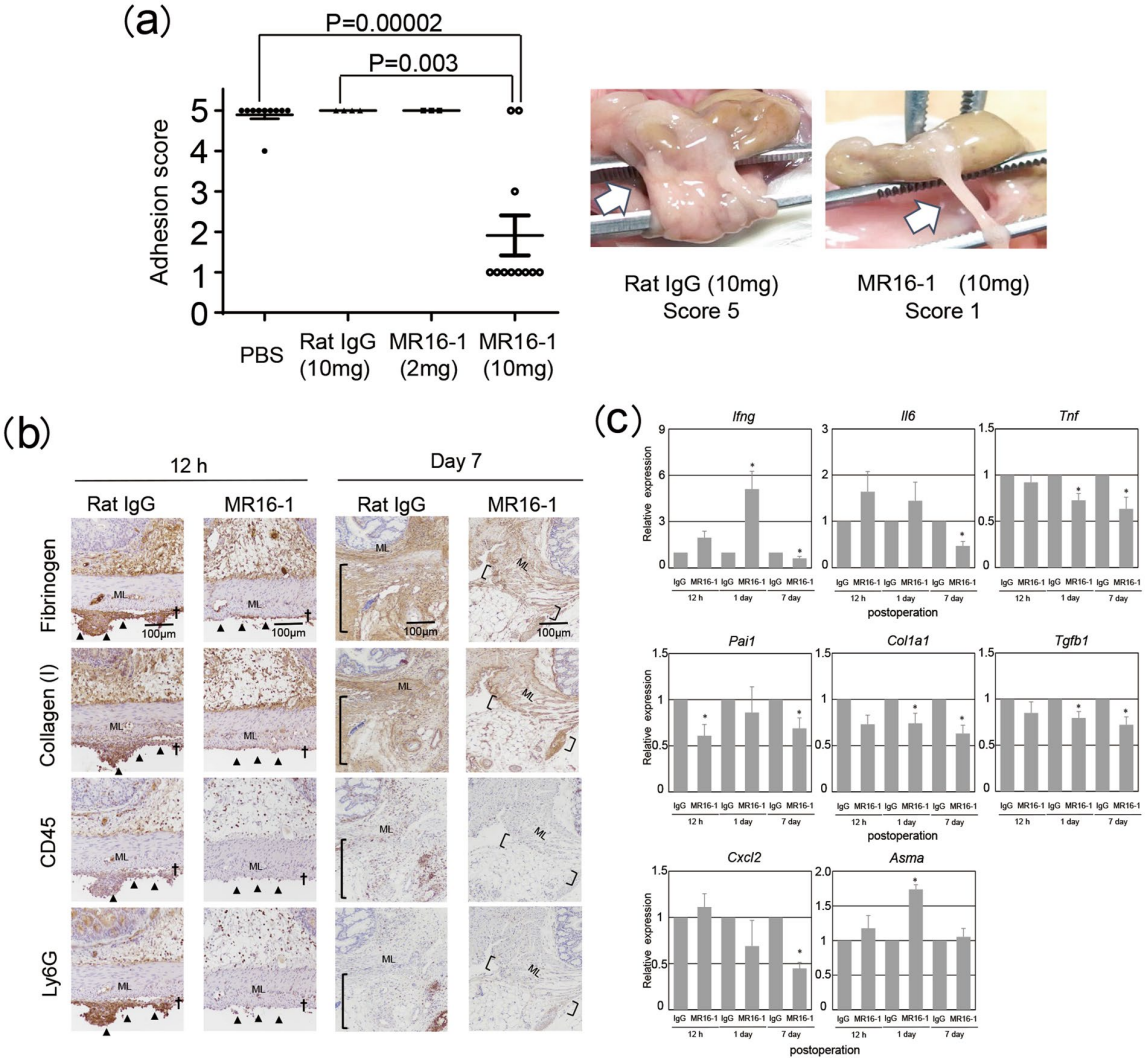
Anti-IL-6Rα antibody protection against postoperative adhesion formation. Wild-type mice were treated with various doses of anti-IL-6Rα antibody or isotype-matched antibody and subjected to cecum cauterization. (**a**) Adhesion scores were determined on day 7 following the procedure. Representative photos are shown. (**b,c**) Cecum lesions were sampled at the indicated time points for indicated protein staining (**b**) and for qRT-PCR analysis of indicated proinflammatory and pro-fibrotic mRNA transcripts (**c**). Each experimental group contained 3–5 mice, with two independent experiments performed. Representative photos are shown. Data at 0 day or hour postoperation indicated those in untreated control mice. Data are shown as mean ± SEM. *p < 0.05 (Student’s paired t-test). *Asma*, gene for αSMA.

IL-6 signaling is associated with tissue fibrosis^25–27^. As IL-6 is produced promptly post operation, we evaluated the role of IL-6 signaling in postsurgical adhesion formation. Anti-IL-6Rα mAb administration significantly reduced adhesion formation (Fig. 5a), underscoring the importance of IL-6 signaling in the process. Blockade of IL-6 signaling reduced fibrin deposition with impaired induction of the pro-coagulating *Pai1* expression (Fig. 5b,c) and dampened collagen deposit with poor induction of pro-fibrotic gene expression (*Col1*A*1* and *Tgfb1)*, concomitant with abrogated induction of *Tnf*, but not of *Ifng*, expression (Fig. 5b,c). Anti-IL-6Rα mAb treatment also diminished infiltration of CD45^+^ hematopoietic cells, including neutrophils (Fig. 5b). The treatment also diminished induction of *Cxcl2* expression (Fig. 5c). Moreover, blockade of IL-6 signaling reduced phosphorylation of Smad2 and STAT3 in the adhesion tissue (Supplementary Fig. 8). However, blockade of IL-6 signaling did not impair in wound healing process (Supplementary Fig. 9). The results suggested that IL-6/STAT3 signaling likely recruits neutrophils to the injured serosa, activating them to produce TGF-β1 via TNF-α induction, eventually resulting in the transdifferentiation and activation of mesothelial cells via activation of the Smad2/3 pathway. These results underscored that IL-6 signaling contributes to postsurgical adhesion formation via recruitment and activation of pro-fibrotic neutrophils. Thus, targeting IL-6 signaling represents a potential strategy for the prevention of postsurgical adhesions.

## Discussion

Postsurgical adhesion formation, an adverse response to laparotomy, can substantially reduce patient quality of life. We previously demonstrated that lymphocytes produce IFN-γ in response to liberated neuropeptide substance P soon after cecal cauterization, contributing to fibrin deposition with robust induction of *Pai1*, eventually resulting in adhesion band formation^7^. Accordingly, *Ifnγ*^−/−^ mice, impaired in *Pai1* induction, are resistant to adhesion formation. The present study revealed several key events underlying the development of the adhesion band. First, we found that myofibroblasts in the adhesion were derived from mesothelial cells. Second, production of IL-6 and of IFN-γ was rapidly induced post operation, followed by induction of TNF-α expression. Third, neutrophils rapidly accumulated at the injury site, producing TGF-β1. Moreover, TNF-α activated neutrophils to produce TGF-β1. Fourth, neutrophil-depleted mice were resistant to adhesion formation. Ultimately, the role of IL-6 signaling was underscored by the findings that anti-IL-6Rα antibodies reduced neutrophil recruitment and protected against adhesion formation.

TGF-β1-producing neutrophils were accumulated in the injured site, which peaked around 6 h. At day 3, neutrophil migration was substantially disappeared. This may implicate that at day 3 cell type other than neutrophils might produce TGF-β1. Indeed, at day 3 major cell type producing TGF-β1 was αSMA^+^ myofibroblasts. *In vitro* study revealed that TNF activated neutrophils but not mesothelial cells to express *TGFB1*. TGF-β1 stimulated both of the cell types to express *TGFB1*. From these observation, we concluded that TGF-β1 produced by TNF-activated neutrophils might, in turn, stimulated mesothelial cells to transdifferentiated into myofibroblasts and to produce robust TGF-β1.

**Figure 6:**
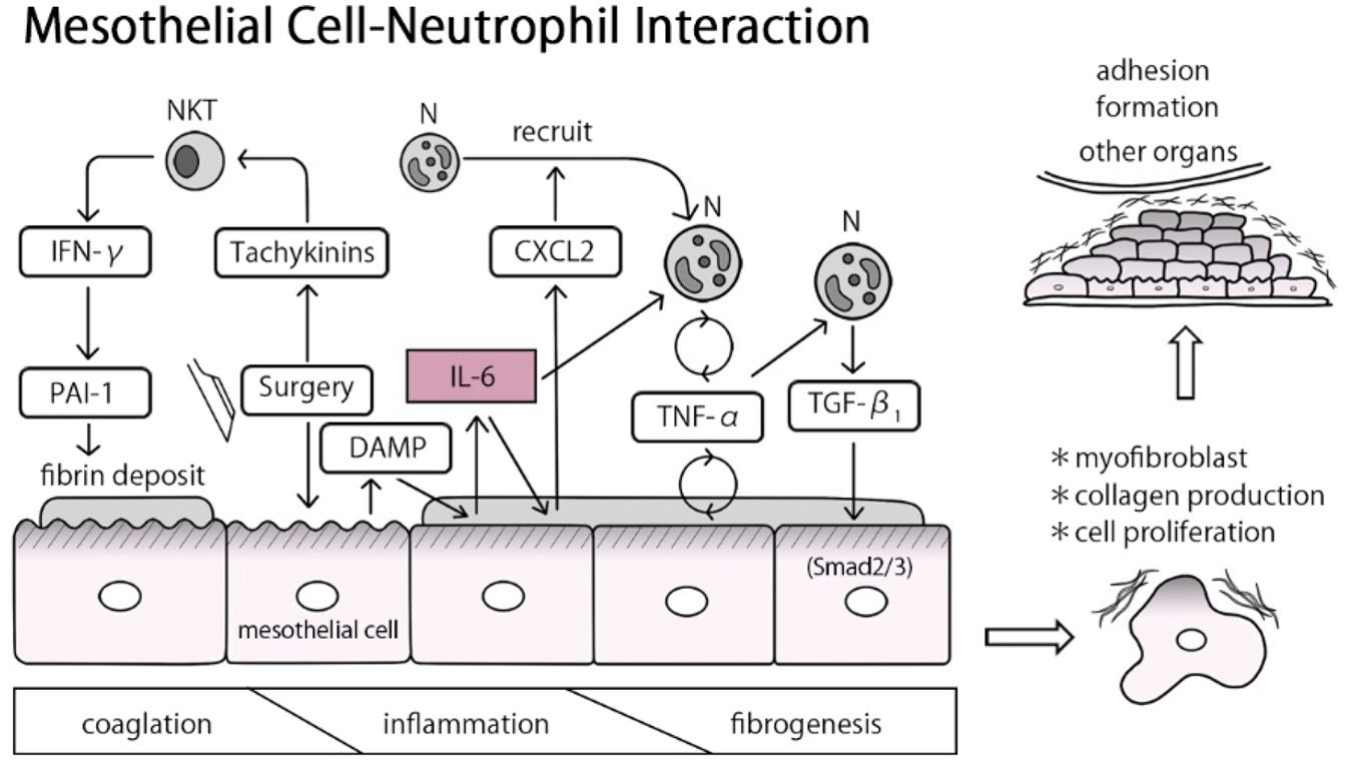
Proposed model for the role of IL-6 in the induction of postoperative adhesion formation. Upon surgery-induced injury, serosa-resident natural killer T (NKT) cells produce IFN-γ, thereby inducing PAI-1, which in turn facilitates fibrin deposition by preventing fibrin degradation as shown previously^7^. Subsequently, mesothelial cells produce CXCL2 and IL-6, which recruit and activate neutrophils, respectively. The neutrophils produce TNF-α in response to IL-6. Both mesothelial cells and neutrophils produce TNF-α in response to TNF-α. Thus, IL-6 signaling plays a central role in activating the TNF-α-amplifying positive-feedback circuits. The final steps of the initiation of adhesion formation occur when TNF-α induces neutrophils, but not mesothelial cells, to produce TGF-β1. TGF-β1 subsequently activates mesothelial cells to transdifferentiate into myofibroblasts that produce fibrosis-promoting molecules such as collagen.

Considering that TGF-β1 has been found to drive the mesothelial-to-mesenchymal transition^23,24,28^, early-stage production of TGF-β1 by neutrophils potentially contributes to the development of the adhesion band. Presently, we underscored the role of mesothelial cells and neutrophils and demonstrated that IL-6 is crucial for the induction of CXCL2, TNF-α, and TGF-β during postoperative adhesion formation (Fig. 6).

TGF-β1 is produced in a latent form lacking biological activity^15,17^. TGF-β1 activation can occur through various biological process including acidification, proteolytic cleavage, and traction by integrin^15,17,29^. Previous reports showed that intraperitoneal injection of mice with monosodium urate monohydrate crystals, a self-derived cause of gout^30^, induces TGF-β1^+^ neutrophils and increases TGF-β1 levels in peritoneal lavage fluid^31^. Notably, human neutrophils increased *TGFΒ1* expression in response to the proinflammatory cytokines IFN-γ and TNF-α. Recently, neutrophil elastase has been shown to activate TGF-β1^32,33^. Neutrophil-elastase-deficient mice are resistant to asbestos- and bleomycin-induced pulmonary fibrosis, which are associated with reduced Smad2/3 phosphorylation^32,33^. Based on these reports and our present findings, we hypothesize that neutrophils recruited to the sites of injury produce active TGF-β1 in response to TNF-α, in turn, facilitating the transdifferentiation and activation of mesenchymal cells into myofibroblasts to enable fibrous adhesion formation. With neutrophil-depleted mice failing to form adhesions, neutrophils likely contribute to the transition from proinflammatory response to pro-fibrotic conditions.

As shown in this study human neutrophils and MeT-5A cells expressed *IL6* in response to TNF-α, IL-6, and TGF-β1 (Fig. 4). The human *IL6* gene includes various *cis*-regulatory elements, including nuclear factor kappa B (NF-κB), CCAAT/enhancer-binding protein beta (C/EBPβ), and interferon regulatory factor 1 (IRF-1), suggesting that wide variety stimuli can induce *IL6*^34^. Indeed, TNF-α has been regarded as potent activator of *IL6* in many cell types^35^. Astrocytes and osteocytes can produce IL-6 in an autocrine manner^36,37^. TGF-β1 was reported to activate cardiac fibroblasts and cervical epithelial cells to produce IL-6^38,39^.

IL-17 has been reported to be a key molecule for the postsurgical adhesion. Neutralizing Abs specific for IL-17 protected against adhesion formation post cecum abration^40^. Cellular source of IL-17 was shown to be T cells, γδT cells and Th17 cells derived from αβT cells, during adhesion formation^41^. Indeed, αβTCR^−/−^ mice that lack αβT cells but possess γδT cells are resistant to the adhesion. Adoptive transfer of WT T cells restored the sensitiveness to the adhesion formation of αβTCR^−/−^ mice, while that of *Stat4*^−/−^ T cells lacking potential to develop into Th1 cells did not^40^. This clearly indicated the importance of Th1 cells as well. Thus, IL-17 may promote adhesion formation and additional stimulation with IFN-γ enhanced this effect. Since IL-6 can differentiates naïve CD4^+^αβT cells into Th17 cells, IL-6 might contribute to the adhesion formation via inducing Th17 cell development as well^34^.

M2, but not M1, macrophages can induce fibrosis by producing signature molecules including TGF-β1. M2 macrophage differentiation occurs upon exposure to type 2 cytokines, such as IL-4 and IL-13^28,42,43^, or upon ingestion of apoptotic cells^44^. IL-13 can induce fibrosis by stimulating human macrophages to produce TGF-β1 via activation of IL-13Rα2, alternatively termed an IL-13 decoy receptor^45^. M2-macrophage-derived TGF-β1 contributes to various type of tissue fibrosis^28,46,47^, including irradiation-induced pulmonary fibrosis^48^, renal fibrosis induced by acute kidney injury^49^, and chemokine (C-C motif) ligand 4-induced liver fibrosis^50^. Thus, macrophages contribute to fibrosis following M2 differentiation. In contrast, human neutrophils can produce TGF-β1 directly in response to the proinflammatory cytokine TNF-α, highlighting their potential role as a key initiator of pro-fibrotic response under inflammation-dominant conditions.

Presently, pathway analysis suggested that activation of pattern recognition receptors occurs upstream of IL-6 signaling. Cauterized serosa release DAMPs, which can potentially activate pattern recognition receptors, including toll-like receptors on innate cells, to induce production of IL-6^51^. Alternatively, DAMPs may induce sterile inflammation by activating the inflammasome in these cells to produce biologically active IL-1β, which may subsequently induce IL-6 production^52,53^.

Tocilizumab, an anti-human IL-6Rα monoclonal antibody, has been used for the treatment of chronic inflammatory diseases, such as rheumatoid arthritis^54,55^. Such IL-6-targeting therapy could also be used as a prophylactic measure for the prevention of postsurgical adhesions.

In this study, we focused on the role of IL-6 on the basis of results of microarray analysis and found the importance of IL-6 signaling for the adhesion formation. However, our present study does not exclude the possibility that other proinflammatory cytokine, in particular TNF, contributes to the adhesion formation as well. Intriguingly, IL-6 blockade protected against the adhesion formation, concomitantly with reduction in induction of *Tnf*. This may provide us with a second hypothesis that TNF signaling might be a key for the development of the adhesion as well. We will test this hypothesis, and if this is the case we will address how importantly IL-6 and TNF signalings contribute to the postsurgical adhesion formation.

## Materials and Methods

### Cell lines

Human mesothelial MeT-5A cells (CRL-9444) and HepG2 cells (HB-8065) were purchased from the American Type Culture Collection. Human mesothelial MES-F cells were from Zen-Bio Inc., and human liver myofibroblast cell line LX-2 cells were donated from Professor Scott Friedman at Mount Sinai School of Medicine. All cell lines were maintained at 37°C and 5% CO_2_ in a humidified atmosphere. MeT-5A cells and MES-F cells were cultured in Medium 199 (Gibco) with 10% FBS (Thermo Scientific). HepG2 cells were cultured in RPMI 1640 (Gibco) with 10% FBS. LX-2 cells were cultured in DMEM (Gibco) with 10% FBS.

### Reagents

Recombinant (r) human (h) TNF-α (21-TA), rhIL-6 (206-IL), and rhTGF-β1 (240-B) were purchased from R& D Systems. rhsIL-6Rα(00–06RC) was obtained from Peprotech. Inc. TaqMan Gene Expression Assays of *Ifng* (Mm01168134_m1), *Il6* (Mm00446190_m1), *Tnf* (Mm00443258_m1), *Pai1* (Mm00435858_m1), *Col1a1* (Collagen type 1α1) (Mm00801666_g1), *Tgfb1* (Mm01178820_m1), *Cxcl2* (Mm00436450_m1), *Asma* (Mm00725412_s1), *IL6* (Hs00174131_m1), *TNF* (Hs00174128_m1), *TGFB1* (Hs00998133_m1), *CXCL2* (Hs00601975_m1), and *COL1A1* (Hs00164004_m1) were purchased from Applied Biosystems. Rat anti-mouse IL-6 Rα antibody (MR16-1) was a gift from Chugai Pharmaceuticals Co. Ltd. and anti-human IL-6Rα monoclonal antibody (mAb) was purchased from Chugai Pharmaceuticals Co. Ltd.

### Mice

We purchased C57BL/6 (B6) mice and Wt1GFPCre mice (010911) from Jackson Laboratory. We bred B6 mice and Wt1GFPCre mice under specific pathogen–free conditions at the animal facilities of Hyogo College of Medicine. All animal experiments were performed in accordance with the guidelines of the Institutional Animal Care Committee, Hyogo College of Medicine.

### Mouse model of surgical adhesion formation

We anesthetized mice with 0.15 ml (20% vol/vol) diluted pentobarbital sodium solution (10 mg/ml) and made an anterior midline incision through the abdominal wall. We isolated the cecum and cauterized it using the coagulation mode of bipolar forceps (MERA; 30 W, 500 kHz, 150 Ω) for one second. We closed the incision in two layers with silk sutures. At the indicated time points post operation, serum, injured ceca, adhesion tissues were sampled. At the indicated time point, we added PBS in peritoneal cavity, and sampled the lavage fluid for peritoneal fluid. At 7 days after operation, adhesion formation in the abdominal cavity was investigated. Each mouse was evaluated according to the following standard scoring system, which has been widely used: score 0, no adhesion; score 1, one thin filmy adhesion; score 2, more than one thin adhesion; score 3, thick adhesion with focal point; score 4, thick adhesion with plantar attachment or more than one thick adhesion with focal point; and score 5, very thick vascularized adhesion or more than one plantar adhesion.

### Human samples

Specimens of adhesion tissues and transudates were collected from the patients during hepatectomies or pancreatectomies at the Hyogo College of Medicine (Nishinomiya, Hyogo, Japan) between March 2018 and November 2018. Ascites and sera were sampled at the indicated time points during and after pancreatectomies at the Hyogo College of Medicine between October 2018 and November 2018. All research protocols of these studies were approved by the Ethics Committee of Hyogo University Graduate School of Medicine (approval number 201805-016, 201810-012). All research was in compliance with the terms of the 1964 Declaration of Helsinki and its later amendments or comparable ethical standards. We obtained informed consent with human subject.

### Immunohistochemistry of human samples

Immunohistochemistry was performed to test whether fibrinogen and collagen type I were present in the adhesion lesions and exudates. Tissue specimens were fixed for 24 h in IHC Zinc fixative solution (BD Biosciences) and embedded in paraffin wax before sectioning. Sections of 4 µm thickness for each specimen were prepared on peeling prevention coated slide glass (Matsunami Glass Ind.) and stained with hematoxylin and eosin (H&E). For immunohistochemistry, the sections were preincubated with serum-free protein block (Agilent Technologies) for 30 min at room temperature and primary antibodies for fibrinogen (ab92572, Abcam) and collagen type I (F56, Kyowa Pharma Chemical Co. Ltd.) diluted in PBS were added to each slide overnight at 4 °C. Secondary antibody staining was performed using the Histofine Simple Stain MAX-PO (Nichirei) according to the manufacturer’s instructions. The staining was visualized under a Nikon Eclipse TS-100 microscope (Nikon, Tokyo, Japan).

### Immunohistochemistry of mouse samples

Immunohistochemistry was performed to investigate the presence of fibrinogen, collagen type I, phosphorylated Smad2 and phosphorylated STAT3, and the accumulation of leucocytes and neutrophils in the injured ceca and adhesion tissues. Tissue specimens were fixed for 48 h in 10% buffered formalin and embedded in paraffin wax before sectioning. For immunohistochemistry, the sections were then preincubated with serum-free protein block (Agilent Technologies) for 30 min at room temperature, after antigen retrieval by incubating slides in 0.01 M Citrate buffer pH 6.0 in 95°C water bath for 30 min. Primary antibodies specific for collagen type I (ab34710, Abcam), fibrinogen (ab27913, Abcam), phosphorylated Smad2 (ab188334, Abcam), phosphorylated STAT3 (ab76315 Abcam), CD3 (18-0102 Invitrogen), B220 (BD Bioscience 550286), F4/80 (BMA Biomedicals T-2006), Ly6G (551459, BD Biosciences), and CD45 (550539 BD Biosciences) were used. Secondary antibody staining was performed using Histofine Simple Stain Mouse MAX-PO (Nichirei) according to the manufacturer’s instructions. The staining was visualized under a Nikon Eclipse TS-100 microscope (Nikon, Tokyo, Japan). In some experiments, we measured histochemical intensity by image analysis using ImageJ^7,8^.

### Immunofluorescent staining with confocal microscopy

For immunofluorescent multiple immunostaining, tissue samples were fixed in 10% buffered formalin and embedded in paraffin wax. In order to investigate cells expressing mesothelial markers in the adhesion tissues, triple fluorescent immunostaining with antibodies for PDPN (ab11936, Abcam), WT-1 (ab89901, Abcam) and α-SMA (ab124964, Abcam) was performed. To investigate proliferation of PDPN-positive cells in damaged ceca and adhesion tissues, double immunostaining was performed with antibodies against PDPN and Ki-67 (#12202 Cell signaling). In order to investigate whether neutrophils and/or mesothelial cells express TGF-β1, IL-6, and/or TNF-α, double immunostaining of TGF-β1, IL-6, and TNF-α in combination with Ly6G or α-SMA was performed. Antibody against for TGF-β1 (ab170874) was purchased from Abcam, and antibodies against for IL-6 (21865-1-AP) and TNF-α (17590-1-AP) were from Proteintech Group Inc. Fluorochrome labeling was performed with Opal 4-color fluorescent IHC kit (NEL820001KT, PerkinElmer) and viewed under a Zeiss LSM780 confocal microscope and documented using LSM780 software.

### Quantitative reverse transcription PCR (qRT-PCR)

We extracted total RNA from the murine tissue samples, MeT-5A cells (human), and human neutrophils with the RNeasy Plus Mini Kit (Qiagen) and synthesized the cDNA using SuperScript III RNase H−Reverse Transcriptase (Invitrogen). We performed qRT-PCR with a StepOnePlus Real-Time PCR System (Applied Biosystems). The results were analysed with StepOne software v.2.0 (Life Technologies). The fold change of each gene expression was analyzed using the 2−ΔΔCt method. According to the 2−ΔΔCt method, each Ct value was first normalized to the internal reference gene (18 S ribosomal RNA, Hs99999901_s1) of the sample and, afterward, to the controls.

### Enzyme-linked immunosorbent assay

We measured the abundance of IL-6, TNF-α, TGF-β1 and sIL-6Rα in sera of mice with mouse ELISA kits of IL-6 (M6000B, R&D Systems), TNF-α (MTA00B, R&D Systems), TGF-β1 (MB100B, R&D Systems), and sIL-6Rα (DY1830, R&D Systems), respectively. The abundance of IL-6 in human sera and ascites was measured by a human ELISA kits of IL-6 (D6050, R&D Systems).

### Microarray analysis

Total RNA was isolated from mouse cecum tissues at 3 h, 12 h and 72 h after cauterization and at 0 h after the sham operation. After fragmentation of complementary RNA, microarray studies were performed using an Agilent SurePrint G3 Mouse GE v2 8 × 60 K Microarray Kit (Design ID 74809, Agilent Technologies). The arrays were scanned using an Agilent SureScan G4900DA (Agilent Technologies). Data analysis was performed with GeneSpring GX 14.9 (Agilent Technologies). Signal intensities for each probe were normalized to the 75th percentile without baseline transformation. Genes that were differentially expressed in comparison to the sham control were selected with the thresholds, which showed a fold change more than 2 and a false discovery rate (FDR) adjusted P-value less than 0.05, for further pathway analysis. All data are MIAME compliant, and the raw data have been deposited in the Gene Expression Omnibus (www.ncbi.nlm.nih.gov/geo, accession no. GSE123413).

### Knowledge-based pathway analysis and multivariate analysis

To explore the biological interpretation of the transcriptome data, pathway analysis was performed using knowledge-based functional analysis software Ingenuity Pathways Analysis (Ingenuity Systems, Mountain View, CA, USA)^56^ as previously described^57^. With details, “Disease and bio function analysis”, “Canonical pathway analysis” and “Upstream regulator analysis” were performed to identify upstream regulatory molecules and associated pathways of the observed expression changes. Upstream regulator analysis is one of the causal analytics algorithms in IPA that was developed to identify the upstream molecules in the data set that can explain the observed expression changes. One of the statistical measures of IPA is the activation z-score, which can be used to find likely regulating molecules based on a statistically significant pattern match of up- and down-regulation, and also to predict the activation state (either activated or inhibited) of a putative regulator. An absolute z-score more than 2 was considered as significant. Unsupervised principle component analysis (PCA) was run using R.

### Human neutrophil isolation and culture

Human neutrophils were isolated from healthy donor blood by the MACSxpress Whole Blood Neutrophil isolation kit (130-104-434, Miltenyi Biotec). The cell viability was evaluated by trypan blue dye exclusion (15-250-061, Gibco). The neutrophils (1 × 10^6^ cells/ml) in RPMI 1640 with 10% FBS were incubated with various recombinant cytokines for 18 h at 37 °C and 5% CO2.

### Neutrophil depletion

Mice were injected intraperitoneally with 500 μg of the monoclonal antibody against anti-Ly6G (Bio X Cell) at 1 day before operation and 2 days and 5 days after operation. Control mice were injected with an equivalent amount of an isotype control IgG2a, clone 2A3 (Bio X Cell). At 7days after operation, adhesion formation was evaluated macroscopically.

### Culture of mesothelial cells

MeT-5A cells (3 × 10^5^ cells were seeded in 3.5 cm dish in in RPMI containing 10% FBS) were incubated with various rh cytokines for 24 h at 37 °C and 5% CO2.

### IL-6 Receptor α blockade

In order to block IL-6 receptor α, MR16-1 (2 or 10 mg) was intraperitoneally injected into mice at 1 day before operation. In both experiments, control mice were injected with an equivalent amount of ChromPure Rat IgG (Bio X Cell).

### Western blotting

Human cells were homogenized in sample buffer (62.5 mmol/l Tris-HCl, pH 6.8, 2% sodium dodecyl sulfate (SDS), 1 mM NaF, 1 mM Na3VO4, and 10% glycerol), and boiled for 10 min. Samples were separated by SDS polyacrylamide gel electrophoresis (5–20% gradient gel; ATTO) and electroblotted onto polyvinylidene difluoride membrane (Millipore). Detection of proteins of interest was performed with specific antibodies and immunoreactive bands were captured using a LAS-3000 imaging system (Fujifilm). Antibodies specific for gp130 (#3732), TNFαR-1 (#3736), PAI-1 (#11907) STAT3 (#12640), phospholyrated-STAT3 (#9145), Smad2/3 (#8685) and phospholylated-Smad2/3 (#8828) were purchased from Cell Signaling. Antibodies specific for collagen type I (ab34710) and αSMA (ab124964) were purchased from Abcam. Antibodies specific for TGF-βR1 (sc-9048) and IL-6Rα (cat.no. sc-661) were purchased from Santa Cruz Biotechnology. Antibody for IFNγR (GTX54333) and β-actin (A1978) was purchased from Gene Tex Inc. and Santa Cruz Biotechnology, Inc. and Sigma-Aldrich., respectively.

### Wound healing

After being anesthetized, backs of mice were shaved, and remnant hairs were removed by depilatory cream (Epilat, Kracie Holdings). Two full-thickness excisional skin wounds were performed on either side of dorsal midline using a disposable sterile 6-mm biopsy punch (Kai Industries, Japan). Each wound was photographed at the indicated time points, and wound area (mm^2^) was calculated with Image software (version 1.48j, NIH).

### Statistical analyses

Data are given as means ± SEM. We performed statistical comparisons between two experimental groups by Student’s paired t-test. Among more than three experimental groups, statistical comparisons were performed by Dunnett’s test. All statistical analyses were performed by using Prism GraphPad Prism5 software (Graphpad Software version 7). We considered a P value < 0.05 as significantly different.

### Ethical approval and informed consent

All animal experiments were performed in accordance with the guidelines of and approved by the Institutional Animal Care Committee, Hyogo College of Medicine. All research protocols for human studies were approved by the Ethics Committee of Hyogo University Graduate School of Medicine (approval number 201805-016, 201810-012). All research was in compliance with the terms of the 1964 Declaration of Helsinki and its later amendments or comparable ethical standards. We obtained informed consent with human subject.

## Supplementary information

supplementary Information

## Data Availability

All microarray data are MIAME compliant, and the raw data have been deposited in the Gene Expression Omnibus (www.ncbi.nlm.nih.gov/geo, accession no. GSE123413 https://www.ncbi.nlm.nih.gov/geo/query/acc.cgi?acc = GSE123413). All data needed to evaluate the conclusions in the paper are present in the paper or the Supplementary Figures.

## References

[1] Holmdahl L (1999). Making and covering of surgical footprints. Lancet.

[2] Cheong YC (2001). Peritoneal healing and adhesion formation/reformation. Human reproduction update.

[3] Diamond MP, Freeman ML (2001). Clinical implications of postsurgical adhesions. Human reproduction update.

[4] Vrijland WW, Jeekel J, van Geldorp HJ, Swank DJ, Bonjer HJ (2003). Abdominal adhesions: intestinal obstruction, pain, and infertility. Surgical endoscopy.

[5] Schnüriger B (2011). Prevention of postoperative peritoneal adhesions: a review of the literature. Am J Sur.

[6] Cesarman-Maus G, Hajjar KA (2005). Molecular mechanisms of fibrinolysis. British journal of haematology.

[7] Kosaka H, Yoshimoto T, Fujimoto J, Nakanishi K (2008). Interferon-gamma is a therapeutic target molecule for prevention of postoperative adhesion formation. Nat. Med..

[8] Ohashi K (2014). Interferon gamma and plasminogen activator inhibitor 1 regulate adhesion formation after partial hepatectomy. The British journal of surgery.

[9] Isaza-Restrepo A, Martin-Saavedra JS, Velez-Leal JL, Vargas-Barato F, Riveros-Duenas R (2018). The Peritoneum: Beyond the Tissue - A Review. Frontiers in physiology.

[10] Hinz B (2007). The myofibroblast: one function, multiple origins. The American journal of pathology.

[11] Hinz B (2012). Recent developments in myofibroblast biology: paradigms for connective tissue remodeling. The American journal of pathology.

[12] Tsai Jonathan M., Sinha Rahul, Seita Jun, Fernhoff Nathaniel, Christ Simon, Koopmans Tim, Krampitz Geoffrey W., McKenna Kelly M., Xing Liujing, Sandholzer Michael, Sales Jennifer Horatia, Shoham Maia, McCracken Melissa, Joubert Lydia-Marie, Gordon Sydney R., Poux Nicolas, Wernig Gerlinde, Norton Jeffrey A., Weissman Irving L., Rinkevich Yuval (2018). Surgical adhesions in mice are derived from mesothelial cells and can be targeted by antibodies against mesothelial markers. Science Translational Medicine.

[13] Cheng Y, Ma XL, Wei YQ, Wei XW (2019). Potential roles and targeted therapy of the CXCLs/CXCR2 axis in cancer and inflammatory diseases. Biochimica et biophysica acta. Reviews on cancer.

[14] Daley JM, Thomay AA, Connolly MD, Reichner JS, Albina JE (2008). Use of Ly6G-specific monoclonal antibody to deplete neutrophils in mice. Journal of leukocyte biology.

[15] Meng XM, Nikolic-Paterson DJ, Lan HY (2016). TGF-beta: the master regulator of fibrosis. Nature reviews. Nephrology.

[16] Fabregat I (2016). TGF-beta signalling and liver disease. Febs J.

[17] Aschner Y, Downey GP (2016). Transforming Growth Factor-beta: Master Regulator of the Respiratory System in Health and Disease. American journal of respiratory cell and molecular biology.

[18] Biancheri P (2014). The role of transforming growth factor (TGF)-beta in modulating the immune response and fibrogenesis in the gut. Cytokine & growth factor reviews.

[19] Holmdahl L (2001). Overproduction of transforming growth factor-beta1 (TGF-beta1) is associated with adhesion formation and peritoneal fibrinolytic impairment. Surgery.

[20] Gorvy DA, Herrick SE, Shah M, Ferguson MW (2005). Experimental manipulation of transforming growth factor-beta isoforms significantly affects adhesion formation in a murine surgical model. The American journal of pathology.

[21] Garbers C, Heink S, Korn T, Rose-John S (2018). Interleukin-6: designing specific therapeutics for a complex cytokine. Nature reviews. Drug discovery.

[22] Lokau J, Agthe M, Flynn CM, Garbers C (2017). Proteolytic control of Interleukin-11 and Interleukin-6 biology. Biochimica et biophysica acta. Molecular cell research.

[23] Sandoval P (2016). Mesothelial-to-mesenchymal transition in the pathogenesis of post-surgical peritoneal adhesions. The Journal of pathology.

[24] Jin X, Ren S, Macarak E, Rosenbloom J (2015). Pathobiological mechanisms of peritoneal adhesions: The mesenchymal transition of rat peritoneal mesothelial cells induced by TGF-β1 and IL-6 requires activation of Erk1/2 and Smad2 linker region phosphorylation. Matrix Biol.

[25] O’Reilly S, Ciechomska M, Cant R, Hugle T, van Laar JM (2012). Interleukin-6, its role in fibrosing conditions. Cytokine & growth factor reviews.

[26] Natsume M (1999). Attenuated liver fibrosis and depressed serum albumin levels in carbon tetrachloride-treated IL-6-deficient mice. Journal of leukocyte biology.

[27] Zhang WR (2012). Interleukin 6 Underlies Angiotensin II-Induced Hypertension and Chronic Renal Damage. Hypertension.

[28] Gieseck RL, Wilson MS, Wynn TA (2018). Type 2 immunity in tissue repair and fibrosis. Nat Rev Immunol.

[29] Wipff P-J, Rifkin DB, Meister J-J, Hinz B (2007). Myofi broblast contraction activates latent TGF-β1 from the extracellular matrix. The Journal of cell biology.

[30] Perez-Ruiz F, Dalbeth N, Bardin T (2015). A review of uric acid, crystal deposition disease, and gout. Advances in therapy.

[31] Steiger S, Harper JL (2013). Neutrophil Cannibalism Triggers Transforming Growth Factor 1 Production and Self Regulation of Neutrophil Inflammatory Function in Monosodium Urate Monohydrate Crystal–Induced Inflammation in Mice. Arthritis and rheumatism.

[32] Chua F (2007). Mice lacking neutrohil elastase are resistant to bleomycin induced pulmonary fibrosis. The American journal of pathology.

[33] Gregory AD (2015). Neutrophil elastase promotes myofibroblast differentiation in lung fibrosis. Journal of leukocyte biology.

[34] Narazaki, M. & Kishimoto, T. The Two-Faced Cytokine IL-6 in Host Defense and Diseases. *International journal of molecular sciences*, **19**(2018). 10.3390/ijms19113528 PMC6274717 30423923

[35] Kishimoto T, Taga T, Akira S (1994). Cytokine signal transduction. Cell.

[36] Van Wagoner NJ, Oh JW, Repovic P, Benveniste EN (1999). Interleukin-6 (IL-6) production by astrocytes: autocrine regulation by IL-6 and the soluble IL-6 receptor. The Journal of neuroscience: the official journal of the Society for Neuroscience.

[37] Franchimont N, Rydziel S, Canalis E (1997). Interleukin 6 is autoregulated by transcriptional mechanisms in cultures of rat osteoblastic cells. The Journal of clinical investigation.

[38] Narikawa M (2018). Acute Hyperthermia Inhibits TGF-beta1-induced Cardiac Fibroblast Activation via Suppression of Akt Signaling. Scientific reports.

[39] Sharkey DJ (2012). TGF-beta mediates proinflammatory seminal fluid signaling in human cervical epithelial cells. J Immunol.

[40] Chung DR (2002). CD4+ T cells regulate surgical and postinfectious adhesion formation. The Journal of experimental medicine.

[41] Wang G (2014). Role of IL-17 and TGF-beta in peritoneal adhesion formation after surgical trauma. *Wound repair and regeneration: official publication of the Wound Healing Society [and] the European Tissue Repair*. Society.

[42] Wynn TA (2015). Type 2 cytokines: methanisms and therapeutics strategies. Nature reviews. Immunology.

[43] Meng XM, Ming-Kuen Tang P, Yao Lan H (2015). Macrophage Phenotype in Kidney Injury and Repair. Kidney Dis.

[44] Fadok VA (1998). Macrophages That Have Ingested Apoptotic Cells *In Vitro* Inhibit Proinflammatory Cytokine Production Through Autocrine Paracrine Mechanisms Involving TGFb, PGE2, and PAF. The Journal of clinical investigation.

[45] Fichtner-Feigl S, Strober W, Kawakami K, Puri RK, Kitani A (2006). IL-13 signaling through the IL-13alpha2 receptor is involved in induction of TGF-b1 production and fibrosis. Nature medicine.

[46] Wynn TA, Vannella KM (2016). Macrophages in Tissue Repair, Regeneration, and Fibrosis. Immunity.

[47] Teodoro Braga, T., Sebastian Henao Agudelo, J. & Oslsen Saraiva Caara, N. Macrophages During the Fibrotic Process: M2 as Friend and Foe. *Front Immunol*, **6**, Article 602 (2015). 10.3389/fimmu.2015.00602 PMC4658431 26635814

[48] Chung SI (2016). IL-13 is a therapeutic target in radiation lung injury. Sci Rep.

[49] Conway B, Hughes J (2012). Cellular orchestrators of renal fibrosis. QJ Med.

[50] Pradere J-P (2013). Hepatic Macrophages But Not Dendritic Cells Contribute to Liver Fibrosis by Promoting the Survival of Activated Hepatic Stellate Cells in Mice. Hepatology.

[51] Braza F, Brouard S, Chadban S, Goldstein DR (2016). Role of TLRs and DAMPs in allograft inflammation and transplant outcomes. Nature reviews. Nephrology.

[52] Imaeda AB (2009). Acetaminophen-induced hepatotoxicity in mice is dependent on Tlr9 and the Nalp3 inflammasome. Journal of Clinical Investigation.

[53] Tsutsui H, Cai X, Hayashi S (2015). Interleukin-1 Family Cytokines in Liver Diseases. Mediators of inflammation.

[54] Bae SC, Lee YH (2018). Comparison of the efficacy and tolerability of tocilizumab, sarilumab, and sirukumab in patients with active rheumatoid arthritis: a Bayesian network meta-analysis of randomized controlled trials. Clinical rheumatology.

[55] Tarp S (2016). Efficacy and safety of biological agents for systemic juvenile idiopathic arthritis: a systematic review and meta-analysis of randomized trials. Rheumatology.

[56] Kramer A, Green J, Pollard J, Tugendreich S (2014). Causal analysis approaches in Ingenuity Pathway Analysis. Bioinformatics.

[57] Qin XY (2017). Transcriptome Analysis Uncovers a Growth-Promoting Activity of Orosomucoid-1 on Hepatocytes. EBioMedicine.

