# Supplementary material for: Anti-interleukin-6 receptor antibody treatment ameliorates postoperative adhesion formation
Source: Sci Rep. 2019 Nov 26;9:17558. doi: 10.1038/s41598-019-54175-1 (PMC6879753; doi:10.1038/s41598-019-54175-1)
Supplement: Supplementary file 1 — supplementary Information [file 41598_2019_54175_MOESM1_ESM.docx]

**Anti-interleukin-6 receptor antibody treatment ameliorates postoperative adhesion formation.**

Naoki Uyama, Hiroko Tsutsui, Songtao Wu, Koubun Yasuda, Etsuro Hatano, Xian-Yang Qin, Soichi Kojima, Jiro Fujimoto.

**Supplementary Data**

(b)

(a)

Supplementary Fig. 1: Subsequent induction of fibrin deposition and fibrosis formation post operation.

(a). Cecum samples were obtained from mice following 72 h or 7 days after cecum cauterization. (b). Laparotomy exudates and tissue sections were sampled from a subject undergoing first-time laparotomy and a subject with an established postoperative adhesion, respectively. The sections were stained with anti-murine (a) or anti-human (b) fibrinogen or collagen type 1 antibodies, as well as with hematoxylin eosin (H-E). Representative photos are shown. Arrows indicate fibrin-positive cells; arrowheads, collagen-type-1-positive cells; daggers, serosa; opened brackets, adhesion lesions; ML, muscle layer.

(d)

(c)

(b)

(a)

Supplementary Fig. 2: Genome-wide analysis of gene expression in cecal cauterization samples.

Transcriptional profiling was performed to analyze transcript abundance patterns in cecum specimens sampled from control mice and from those at the indicated time points following cecum cauterization. (a). A global view of the dynamic gene expression changes. (b). Canonical pathway analysis was performed using the transcriptomic data. (c). Upstream regulation analysis was performed using the transcriptomic data. (d). IL-6-dependent proposed regulatory network generated by knowledge-based pathway analysis in the IPA platform.

Supplementary Fig. 3: Changes in cytokine levels in the peritoneal fluid following operation.

Peritoneal fluid was sampled from mice at the indicated time points after surgery, followed by measurement of IL-6, TNF-α, TGF-β1, and soluble IL-6Rα (sIL-6Rα) levels using ELISA. Each experimental group contained 3-5 mice. Data are shown as mean ± SEM.

400 µm

400 µm

400 µm

Supplementary Fig. 4: Sparse accumulation of T cells, B cells, and macrophages

Ceca were sampled from mice at 6 h after surgery or control mice. The sections were stained with anti-CD3, anti-B220, and anti-F4/80 antibodies to detect T cells, B cells, and macrophages, respectively. Representative photos are shown.

Supplementary Fig. 5: MeT5A cells express receptors for various cytokines.

MeT-5A (human pleural mesothelial cell line), MES-F (immortalized human peritoneal mesothelial cell line, MES), LX-1 (human stellate cell line), and human peripheral blood neutrophils were analyzed for their expression of IFN-γR, TNF-αR1, collagen type 1α1, TGF-β1R, gp130 (signaling receptor for IL-6), IL-6Rα (binding receptor for IL-6), αSMA, and PAI-1, by western blotting. Unprocessed original images of western blots are presented in Supplementary Fig. 13.

(b)

(a)

Supplementary Fig. 6: Both neutrophils and mesotherial cells produce TNF-α after surgery.

Cecum specimens were sampled from control mice and animals that underwent cecum cauterization 3 h or 3 days following the procedure. The slides were stained for Ly6G (neutrophils) (a) or αSMA (myofibroblasts) (b), and TNF-α (a, b). Representative photos are shown. Arrows indicate representative Ly6G^+^TNF-α^+^ neutrophils (a) and αSMA^+^ TNF-α^+^ myofibroblasts (b). Daggers indicate serosa; open brackets indicate adhesion lesions.

(a)

(b)

Supplementary Fig. 7: Both neutrophils and mesotherial cells produce IL-6 after surgery.

Cecum specimens were sampled from control mice and animals that underwent cecum cauterization 3 h or 3 days following the procedure. The slides were stained for Ly6G (neutrophils) (a) or αSMA (myofibroblasts) (b), and IL-6 (a, b). Representative photos are shown. Arrows indicate representative Ly6G^+^IL-6^+^ neutrophils (a) and αSMA^+^IL-6^+^ myofibroblasts (b). Daggers indicate serosa; opened brackets indicate adhesion lesions.

Supplementary Fig. 8: Effects on STAT3 and Smad2 phosphorylation of treatments with anti-IL-6Rα antibodies (MR16-1, right panels) and control IgG (left panels).

Representative photos are shown.

(b)

(a)

Supplementary Fig.9. Intact wound healing in mice treated with anti-IL-6R mAb.

Wild-type mice that had been treated with anti-IL-6R mAb (n=4) or control rat IgG (n=4) 1 day before were injured by cutaneous biopsy. The repair rate of the wound areas was monitored and shown by mean ± SEM (a). Representative photos are shown (b).

Supplementary Fig.10. Unprocessed original images of western blots related to Supplementary Fig.5.
